# Oral treatment with *Lactobacillus rhamnosus* attenuates behavioural deficits and immune changes in chronic social stress

**DOI:** 10.1186/s12916-016-0771-7

**Published:** 2017-01-11

**Authors:** Aadil Bharwani, M. Firoz Mian, Michael G. Surette, John Bienenstock, Paul Forsythe

**Affiliations:** 1 Department of Pathology & Molecular Medicine, McMaster University, Hamilton, Canada; 2 McMaster Brain-Body Institute, The Research Institute of St. Joseph’s Hamilton, Hamilton, Canada; 3 Michael G. DeGroote School of Medicine, McMaster University, Hamilton, Canada; 4 Department of Medicine, McMaster University, The Brain-Body Institute, 50 Charlton Avenue East, T3302, Hamilton, Ontario L8N 4A6 Canada; 5 Farncombe Family Digestive Health Research Institute, McMaster University, Hamilton, Canada; 6 Firestone Institute for Respiratory Health, St. Joseph’s Healthcare Hamilton, Hamilton, Canada

**Keywords:** Chronic social defeat, Microbiota, Behaviour, Immune system, Gut-brain axis, Psychiatric disease

## Abstract

**Background:**

Stress-related disorders involve systemic alterations, including disruption of the intestinal microbial community. Given the putative connections between the microbiota, immunity, neural function, and behaviour, we investigated the potential for microbe-induced gut-to-brain signalling to modulate the impact of stress on host behaviour and immunoregulation.

**Methods:**

Male C57BL/6 mice treated orally over 28 days with either *Lactobacillus rhamnosus* (JB-1) ™ or vehicle were subjected to chronic social defeat and assessed for alterations in behaviour and immune cell phenotype. 16S rRNA sequencing and mass spectrometry were employed to analyse the faecal microbial community and metabolite profile.

**Results:**

Treatment with JB-1 decreased stress-induced anxiety-like behaviour and prevented deficits in social interaction with conspecifics. However, JB-1 did not alter development of aggressor avoidance following social defeat.

Microbial treatment attenuated stress-related activation of dendritic cells while increasing IL-10+ regulatory T cells. Furthermore, JB-1 modulated the effect of stress on faecal metabolites with neuroactive and immunomodulatory properties. Exposure to social defeat altered faecal microbial community composition and reduced species richness and diversity, none of which was prevented by JB-1.

Stress-related microbiota disruptions persisted in vehicle-treated mice for 3 weeks following stressor cessation.

**Conclusions:**

These data demonstrate that despite the complexity of the gut microbiota, exposure to a single microbial strain can protect against certain stress-induced behaviours and systemic immune alterations without preventing dysbiosis. This work supports microbe-based interventions for stress-related disorders.

**Electronic supplementary material:**

The online version of this article (doi:10.1186/s12916-016-0771-7) contains supplementary material, which is available to authorized users.

## Background

Stress-related disorders have their roots in nuanced interactions between genetic and environmental risk factors, resulting in complex and multifactorial etiologies. The cumulative physiological effect of stressors [1] causes the dysregulation of multiple host systems due to allostatic overload. The last decade has witnessed a growing interest in the potential contribution of gut-brain signalling to psychiatric disorders. Chronic severe stress is associated with inflammation and increased susceptibility to functional gastrointestinal conditions, and there is strong evidence for co-morbidity between gastrointestinal symptoms and psychiatric disorders [2–4]. Although precise biological mechanisms remain unclear, it is possible that such bidirectional associations in stress are at least partly a consequence of alterations in gut-brain signalling pathways due to a disrupted gut microbial community. The latter is complex and dynamic, harbouring ~10^13^ bacterial cells that represent 3.3 million non-redundant genes, rivalling our human genome by at least two orders of magnitude [5]. The critical role of the gut microbial community in the regulation of diverse physiological functions, including immunity, is well established, and there is growing evidence of its influence on the central nervous system [6–8]. For instance, administration of specific bacterial strains decreases anxiety- and depressive-like behaviours [9, 10], while changes in the microbial community modulate stress-induced inflammation [11, 12]. The emergent corollary demonstrates the inextricable relationship between the microbiota, immune, and nervous systems, and their roles in regulating behaviour and neural function. Indeed, along with other groups, we have demonstrated the top-down effect of psychological stress on the structure and function of the microbiota, resulting in reduced species diversity and richness, an altered community profile, and shifts in functional pathways [12–14]. Given microbial regulation of host signalling at the mucosal interface between microbiota and host, disruptions in this community may lead to systemic changes in peripheral signals [15, 16]. For instance, immune dysregulation has been implicated in psychological stressors and psychiatric disorders [12, 17]. However, much remains to be determined regarding how bottom-up signalling along the gut-brain axis might be utilized to modulate stress-related changes in behaviour and neural function.

The aim of the present study was to investigate the role of microbe-induced gut-to-brain signalling on the central and systemic disruptions induced by chronic exposure to a psychosocial stressor. Using a validated model of chronic stress and depression [18, 19], we determined whether oral administration of a bacterium with neuroactive and immunomodulatory properties could modulate stress-induced behavioural deficits, immune changes, and gut dysbiosis. We selected *Lactobacillus rhamnosus* JB-1^TM^ (JB-1) as our test organism, as oral treatment with this strain was previously demonstrated to lead to changes in neurotransmitter levels in the brains of mice [20] and to have anxiolytic and anti-depressant-like activity on baseline behaviours—effects dependent on an intact vagus nerve [10]. Feeding the JB-1 strain also modulates enteric nervous system function [21], increases the frequency of vagal afferent firing [22], and has well-described anti-inflammatory and immunoregulatory effects [23–25]. To elucidate metabolites that may drive effects of bacteria on the brain, we investigated candidate functional pathways using metabolomics profiling. Furthermore, we examined the duration of stress-induced disruptions in the microbiota and the possibility of whether administration of a single bacterial strain during stress exposure can facilitate recovery of the dysbiotic community.

## Methods

### Animals

Male C57BL/6 mice, 8 weeks old, and CD-1 retired breeders were acquired from Charles River (Montreal, Canada). Animals were acclimatized for 7 days in standard conditions (12-h light-dark cycle) with *ad libitum* access to standard chow and water. All experiments followed Canadian Council on Animal Care guidelines and were approved by the McMaster Animal Research Ethics Board.

### Treatment and social defeat

**Fig. 1:**
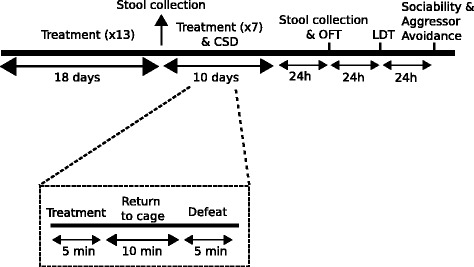
Schematic diagram of experimental approach. Mice were treated with JB-1 on 20 instances over a period of 28 days, including 7 instances over the final 10 days, during which mice were exposed to chronic social defeat (*CSD*) stress every day. *OFT* open field test, *LDT* light-dark box test

Animals were gavaged with 200 μl (1.67 × 10^9^ CFU) of *Lactobacillus rhamnosus* (JB-1) ™—a gift from Alimentary Health Ltd., Cork, Ireland—or equivalent volume of phosphate-buffered saline (PBS). Treatment was administered over 28 days, Monday to Friday, between 1 to 3 p.m. During the final 10 days of treatment (Fig. 1), chronic social defeat (CSD) was initiated as previously described (Additional file 1) [18]. For 24 h after each defeat, mice were housed in the same cage across a perforated Plexiglas divider from their aggressors.

### Behavioural testing

Details of the behavioural testing are provided in Additional file 1. Behavioural testing began 24 h after the final defeat session, and was recorded/analysed using Motor Monitor (Kinder Scientific) and EthoVision XT (Noldus). Anxiety-like and exploratory behaviours were assessed using the light-dark box test (LDT) and open field test (OFT). Sociability and susceptibility were assessed using the three-chamber sociability and aggressor approach-avoidance tests.

### Tissue analysis

Mice were euthanized 5 days after the final defeat session. Spleens were harvested and dispersed using a cell strainer in cold, sterile PBS. Cell suspensions were centrifuged at 1500 rpm for 10 min at 4 °C, then re-suspended in red blood cell (RBC) lysis buffer for 1–2 min. The resulting solution was centrifuged before the cell pellets were washed with 5 ml of complete RPMI 1640 medium: 10% foetal bovine serum, penicillin/streptomycin antibiotics, 2 mM l-glutamine, and 0.01% β-mercaptoethanol. Viable cell numbers were assessed by Trypan Blue exclusion and diluted in RPMI to a concentration of 10^7^ cells/ml. Splenocytes (10^6^) were stained for markers of dendritic cell (DC) maturation and function—CD11c- PerCP-Cy5, MHCII-FITC, CD80-PE, and CD86-APC—or regulatory T cells—CD3-APC, CD4-FITC, CD25-PE-Cy7, and intracellular IL-10-PE (BD Pharmingen, San Diego, CA, USA; eBioscience, San Diego, CA, USA). Following surface staining, cells were fixed and permeabilized with BD Cytofix/Cytoperm before staining for intracellular markers. Data were acquired with FACSCanto (Becton Dickinson, Oakville, ON, Canada) and analysed using FlowJo (TreeStar, Ashland, OR, USA).

### RNA extraction and RT-qPCR analyses

Following rapid decapitation, the frontal cortex and hippocampus were macrodissected using their stereotaxic coordinates according to the Mouse Brain Atlas and placed into RNA*later®* solution (Ambion, Life Technologies, CA, USA). Tissues were incubated overnight at 4 °C and then transferred to –20 °C storage to await further processing. RNA extraction was carried out using TRIzol® Reagent (Ambion, Life Technologies) following manual homogenization. RNA quality was assessed using a NanoDrop*®* Spectrophotometer ND-1000. 1 μg RNA was then converted into complementary DNA (cDNA) by using SuperscriptIII™ First-Strand Synthesis Supermix (Invitrogen, CA, USA). Diluted or non-diluted cDNA was used as a template for qPCR reaction using PowerUp™ SYBR®Green Master Mix (Applied Biosystems, Life Technologies, Austin, TX, USA) containing ROX™ Passive Reference Dye. The qPCR reactions were performed in the fast mode (uracil-DNA glycosylase [UDG] activation 50 °C, 2 min; Dual-Lock™ DNA polymerase 95 °C, 2 min; denaturation 95 °C, 1 s; annealing/extension 60 °C, 30 s; number of cycles: 40) by using QuanStudio3™ (Applied Biosystems). Primers were designed with Primer Express™ Software and used at a concentration of 300 nM. Primer sequences are listed in Additional file 2: Table S6. Transcripts were normalized to endogenous glyceraldehyde-3-phosphate dehydrogenase (GAPDH) and quantified using the ΔΔCt method, with related fold change expressed as 2^(-ΔΔCt)^

### 16S rRNA analysis and metabolomics

Faecal pellets were stored at –80 °C. DNA extraction was carried out as previously described [13]. Bacterial community profiling of 16S rRNA was carried out on a MiSeq Illumina sequencer in the McMaster Genome Center (McMaster University). Metabolite profiling was performed by Metabolon, Inc.

Using rarefied data in QIIME [26], Chao1 and phylogenetic diversity metrics were implemented, and Jackknife resampling was used to generate Bray-Curtis distances. (Dis)similarity between the groups was calculated using the Monte Carlo permutation procedure (MCPP) (999 permutations) and a priori Bonferroni-corrected non-parametric *t* tests. Kruskal-Wallis one-way analysis of variance (ANOVA) or the Mann-Whitney U test, followed by the Benjamini-Hochberg correction for multiple comparisons (false discovery rate, FDR < 0.05), was used to analyse differential abundance of operational taxonomic units (OTUs) in groups.

### Statistical analysis

Data were analysed in IBM’s SPSS (version 22, Chicago) and GraphPad Prism 6 using a two-tailed Student’s *t* test, Mann-Whitney U test, or ANOVAs, with Bonferroni-corrected post hoc tests. Two-way ANOVAs with contrasts followed by Benjamini-Hochberg correction (FDR <0.1) were used to analyse metabolomics data. No statistical methods were used to predetermine sample sizes; however, *n* values used herein are consistent with previous work. During the course of social defeat and testing, some animals were removed due to excessive wounding (open wounds exceeding 1 cm, as per the Animal Utilization Protocol approved by McMaster’s Animal Research Ethics Board). Results in figures are expressed as mean ± standard error of the mean (SEM), where applicable. Statistical significance is denoted as * (*p* < 0.05), ** (*p* < 0.01), and *** (*p* < 0.001).

## Results

### Microbial treatment modulates specific stress-induced behavioural deficits

Chronic social defeat (CSD) reveals distinct phenotypes—susceptible and resilient—based on behaviour in the aggressor approach-avoidance test [18, 19, 27]. CSD induced expression of both phenotypes in either treatment group, with no difference in the proportion of resilient mice: 18.1% (6/33) of vehicle-treated defeated mice and 15.6% (5/32) of defeated mice treated with JB-1 until CSD cessation. Only the susceptible group was used for all experiments.

**Fig. 2:**
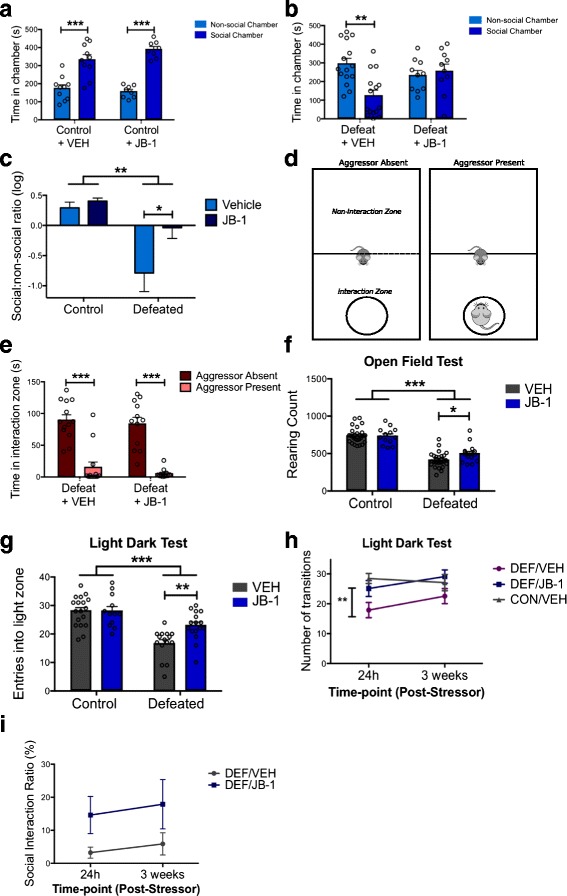
Chronic stress induces deficits in social and anxiety-like behaviours in mice that are partially corrected by microbial treatment. **a** Microbial treatment does not alter baseline sociability, measured by time spent in the social and non-social chambers, in vehicle-treated (*n* = 10) versus JB-1-treated (*n* = 8) unstressed mice. **b** Vehicle-treated defeated mice (*n* = 15) exhibit avoidance of the social chamber—deficits that are corrected in defeated mice treated with JB-1 (*n* = 10). **c** Data demonstrating the time spent in the social and non-social chambers as a log ratio across all four groups. **d** Aggressor approach-avoidance test paradigm. **e** Socially defeated mice exhibit avoidance of a novel aggressor, independent of treatment. **f** Chronic stress reduced rearing behaviour on the OFT, but was partially rescued by JB-1 treatment (*n*: CON/VEH = 29, CON/JB-1 = 13, DEF/VEH = 27, DEF/JB-1 = 16). **g** Chronic stress reduced the number of entries into the light zone on the LDT, but was partially rescued by JB-1 treatment (*n*: CON/VEH = 18, CON/JB-1 = 12, DEF/VEH = 15, DEF/JB-1 = 16). **h** Anxiety-like behaviour across time, as measured by the number of entries into the light zone, at 24 h and at 3 weeks following cessation of CSD treatment (*n*: CON/VEH = 11, DEF/VEH = 9, DEF/JB-1 = 9). **i** Avoidance behaviour on the aggressor approach-avoidance test, at 24 h and at 3 weeks following cessation of CSD treatment (*n*: DEF/SAL = 9, DEF/JB-1 = 10). *p < 0.05, **p <0.01, and ***p < 0.001. Data are represented as mean ± SEM

We have previously demonstrated that mice subjected to CSD exhibit sociability deficits [13]. Vehicle-treated defeated mice (DEF/VEH) exhibited pronounced avoidance of the social chamber (group × chamber interaction [*F*_1, 23_ = 5.438, *p =* 0.029, post hoc, *p* < 0.05]) (Fig. 2b). However, defeated mice administered JB-1 (DEF/JB-1) demonstrated no preference between the social and non-social chambers (post hoc, *p* > 0.05) (Fig. 2b) and, relative to DEF/VEH, exhibited a greater social:non-social ratio (*F*_1, 39_ = 9.660, *p =* 0.004, post hoc, *p* < 0.05) (Fig. 2c), indicating a partial correction of stress-induced deficits in social behaviour. Notably, treatment did not alter baseline behaviour (Fig. 2a).

Susceptible mice markedly avoid interactions with a novel aggressor [18]. Thus, we investigated whether the positive effects of JB-1 extended to behaviour on the aggressor approach-avoidance test (Fig. 2d). DEF/JB-1 mice continued to exhibit pronounced avoidance of the zone surrounding the aggressor (‘interaction zone’) exclusively during the presence of the aggressor (*F*_1, 23_ = 130.8, *p* < 0.0001) (Fig. 2e).

Chronic social stress also induces anxiety-like behaviour and deficits in exploration [13, 28]. On the OFT, stress decreased rearing behaviour (*F*_1, 81_ = 131.2, *p* < 0.0001), indicating reduced exploration. Simple effects analysis of defeated groups revealed that JB-1 significantly attenuated deficits in rearing (*F*_1, 14_ = 6.888, *p* = 0.02) (Fig. 2f). Overall, there was no main effect of treatment on rearing or locomotion. Neither stress exposure nor treatment influenced time spent in the center of the open field (Additional file 1: Figure S1B). On the LDT, both defeated groups exhibited fewer transitions into the light compartment (*F*_1, 57_ = 36.34, *p* < 0.0001), which is a more salient measure of anxiety-like behaviour [29] (Fig. 2 g). However, DEF/JB-1 mice ventured into the light compartment more frequently than DEF/VEH mice, indicating an anxiolytic-like effect of JB-1 administration (stress exposure × treatment interaction [*F*_1, 57_ = 5.171, *p* = 0.027, post hoc, *p* < 0.05]). Neither stress nor treatment affected time spent in the light compartment (Additional file 3: Figure S1C).

Given the paucity of literature regarding the long-term ramifications on behaviour following cessation of interventions, we re-tested a subset of mice 3 weeks following CSD exposure and treatment cessation. Entries into the light compartment 24 h following the final defeat were significantly different between the CON/VEH and DEF/VEH groups, but not between the CON/VEH and DEF/JB-1 or DEF/VEH and DEF/JB-1 groups (*F*_1, 26_ = 6.738, *p* = 0.004, post hoc, CON/VEH versus DEF/VEH at 24 h, *p* < 0.01), further corroborating the anxiolytic-like effects of JB-1 (Fig. 2 h). Three weeks post-stressor, there were no significant differences between any of the three groups, indicating a recovery of stress-induced anxiety-like behaviour. Neither JB-1 nor time influenced aggressor avoidance behaviour 3 weeks post-stressor (Fig. 2i).

To investigate the neural mechanisms underlying the effect of microbial treatment on the expression of stress-related behaviours, we examined changes in expression of genes related to the stress circuitry in the frontal cortex and hippocampus. Neither stress nor treatment altered the expression of corticotropin-releasing factor receptor type 1 or type 2 in the frontal cortex or the hippocampus, or the glucocorticoid receptor in the frontal cortex (Additional file 4: Figure S2A–E). Stress decreased the expression of glucocorticoid receptors in the hippocampus (*F*_1, 16_ = 10.67, *p* = 0.005)—an effect that was not influenced by JB-1 treatment (Additional file 4: Figure S2F). Given that we have previously demonstrated the effects of JB-1 administration on central gamma-aminobutyric acid (GABA) receptors [10], we examined whether similar changes might underlie the effects of the bacteria in a chronic stress model. Stress reduced the expression of GABA_Aα2_ (*F*_1, 30_ = 6.126, *p* = 0.019) and GABA_B1b_ mRNA (*F*_1, 30_ = 5.961, *p* = 0.021) in the frontal cortex, in the absence of a treatment effect (Additional file 4: Figure S2G, H). There were no effects of either stress or treatment on GABA_Aα2_ or GABA_Aα2_ mRNA levels in the hippocampus (Additional file 4: Figure S2I, J).

These data demonstrate that microbial treatment partially corrects the adverse effects of stress on social preference, exploration, and anxiety-like behaviours.

### Microbial treatment regulates stress-induced alterations in the immune phenotype

**Fig. 3:**
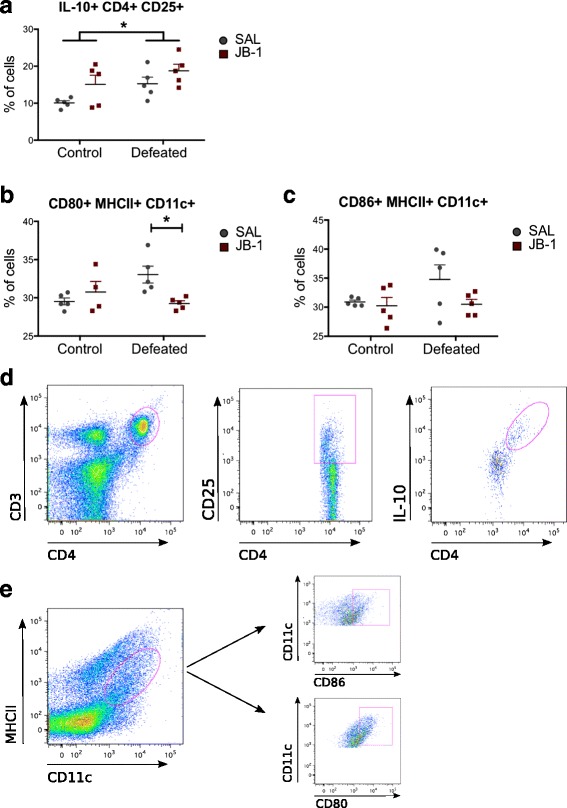
Effect of chronic social defeat stress and JB-1 treatment on splenocyte phenotype (*n* = 5/group). **a** IL-10+ CD4+ CD25+ T cells in mice following exposure to chronic social defeat and JB-1 treatment. **b** JB-1 treatment prevents the stress-induced increase in CD80+ MHCII+ CD11c + splenocyte levels in defeated mice **c** CD86+ MHCII+ CD11c + splenocytes in mice following exposure to chronic social defeat and JB-1 treatment. **d** Fluorescence-activated cell sorting (*FACS*) gating strategy for IL-10+ CD4+ CD25+ T cells (CD3+). **e** FACS gating strategy for CD80+ and CD86+ on MHCII+ DCs (CD11c+). *p < 0.05. Data are represented as mean ± SEM

The immune system represents an important interface for bacteria-host signalling and has been hypothesized as a potential effector of gut-brain communication [7, 15]. CSD increased the population of IL-10+ CD4+ CD25+ T cells (CD3+) (*F*_1, 16_ = 6.114, *p* = 0.025) (Fig. 3a). Microbial treatment alone similarly increased the population of these spleen-derived IL-10-expressing Tregs (*F*_1, 16_ = 5.621, *p* = 0.031). The immunomodulatory effects of JB-1 were not limited to adaptive immunity, as treatment also prevented the stress-induced increase in spleen-derived dendritic cells (MHCII+ CD11c+) expressing markers of activation, CD80 (stress exposure × treatment interaction [*F*_1, 15_ = 8.224, *p* = 0.012, post hoc, *p* < 0.05]) and CD86, though the latter did not reach statistical significance (Fig. 3b, c).

This suggests that administration of JB-1 promoted systemic changes in the immunoregulatory phenotype and influenced the effects of chronic stress on host immunity.

### Microbial treatment does not prevent stress-induced dysbiosis of the microbiota

Multiple groups have confirmed that stress induces dysbiosis [12, 13, 30], correction of which can impart positive effects on the host [31, 32]. Thus, we investigated whether JB-1 exerted its neurobehavioural effects on the stressed host by restoring the microbiota.

**Fig. 4:**
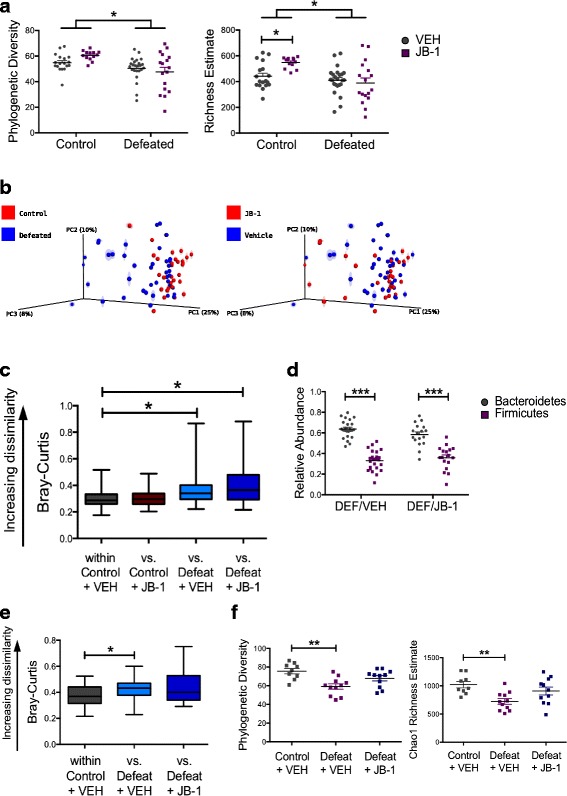
JB-1 treatment does not affect stress-induced structural changes in the microbiota community. **a** Effect of chronic social defeat and JB-1 treatment on phylogenetic diversity and Chao1 richness estimates from the rarefied 16S rRNA data (*n*: CON/VEH = 18, CON/JB-1 = 13, DEF/VEH = 24, DEF/JB-1 = 17, 7923 reads/sample). **b**, **c** Principle coordinates analysis (*PCoA*) of Bray-Curtis distances from the average rarefied 16S rRNA data (*n*: CON/VEH = 18, CON/JB-1 = 13, DEF/VEH = 24, DEF/JB-1 = 17; *n* = 999 rarefactions, 6339 reads/sample) indicate a significant effect of social defeat on group clustering (*p* = 0.013), but no effect of JB-1 treatment (median ± min/max). **d** Effect of chronic social defeat and JB-1 treatment on the taxonomic distribution of OTUs at the phylum level (*n* = 17–24/group). **e** Bray-Curtis distances from the average rarefied 16S rRNA data (*n* = 999 rarefactions, 44,648 reads/sample) three weeks after stressor and treatment cessation indicate a persistent significant effect of social defeat on group clustering (*p* = 0.022), but no difference between the control group and defeated mice treated with JB-1 (median ± min/max). **f** Phylogenetic diversity and Chao1 richness estimates from the rarefied 16S rRNA data (55,810 reads/sample) 3 weeks after stressor and treatment cessation indicate a persistent significant effect of social defeat, but no difference between the control group and defeated mice treated with JB-1 (*n* = 9–11/group). *p < 0.05, **p <0.01, and ***p < 0.001. Data are represented as mean ± SEM unless otherwise indicated

Prior to social defeat, although significantly greater levels of *L. rhamnosus* cells per gram of faeces were detected in mice administered JB-1 (Additional file 3: Figure S1D), JB-1 did not significantly alter the overall profile of the microbial community (Additional file 3: Figure S1E) or post-defeat body weight across groups. As previously described [13], exposure to CSD reduced the diversity (*F*_1, 68_ = 13.21, *p* = 0.0005) and richness (*F*_1, 68_ = 12.50, *p* = 0.0007) of the microbiota (Fig. 4a). These alterations were not ameliorated in DEF/JB-1 mice (post hoc, *p* > 0.05). Assessment of community richness did reveal a significant stress exposure × treatment interaction: CON/JB-1 mice had a richer gut microbiota relative to CON/VEH mice (*F*_1, 68_ = 5.616, *p* = 0.021, post hoc, *p* < 0.05). However, there was no effect of treatment in the defeated groups (post hoc, *p* > 0.05).

To compare group differences in the overall microbiota profile, Bray-Curtis distances (Fig. 4b) were analysed using a priori planned comparisons. Stress altered the microbiota profile: distances within non-defeated mice were smaller than distances between defeated and non-defeated mice (Fig. 4c; Additional file 2: Table S1; Bonferroni-corrected non-parametric *p* = 0.013). JB-1 treatment did not prevent stress-induced changes in the microbiota: profiles within a group (within-DEF/VEH and within-DEF/JB-1) were not significantly closer than profiles from the opposing group (DEF/VEH versus DEF/JB-1), indicating a lack of clustering due to treatment (Bonferroni-corrected non-parametric *p* > 0.05). Moreover, JB-1- and vehicle-treated non-defeated mice formed a separate cluster from DEF/JB-1 mice (Bonferroni-corrected non-parametric *p* = 0.013).

We investigated whether microbial treatment restored the relative abundance of specific OTUs that discriminated defeated mice from the non-defeated controls. Eighteen OTUs (11 Bacteroidetes, 6 Firmicutes, 1 Proteobacteria) were altered by stress exposure (*q* < 0.05) (Additional file 2: Table S2), none of which were restored by JB-1 treatment.

Alterations in the major microbial phyla—Firmicutes and Bacteroidetes—are associated with dysbiosis and disease [33–36]. However, there was no effect of treatment on the Bacteroidetes/Firmicutes ratio (Fig. 4d). Together, these data suggest that JB-1 treatment failed to prevent stress-induced alterations to the microbiota community.

### Stress-induced dysbiosis persists for at least 3 weeks

There is growing evidence from human reports indicating co-morbidity between psychiatric conditions such as depression and post-traumatic stress disorder (PTSD) and gastrointestinal disorders, which are associated with persistent dysbiosis [37]. Thus, 3 weeks following the cessation of CSD, we examined the endurance of stress-induced microbial disruptions and the possibility of whether treatment facilitated recovery.

Group differences due to stress exposure were still evident at this time point (analysis of similarities [ANOSIM], R = 0.1307, *p* = 0.009). Within-group distances in CON/VEH were smaller than the distances versus DEF/VEH mice, indicating separation of CON/VEH and DEF/VEH groups due to stress exposure (Fig. 4e; Additional file 3: Figure S1F; Additional file 2: Table S1 [Bonferroni-corrected non-parametric *p* = 0.022]). There was no statistically significant difference between vehicle- and JB-1-treated defeated mice.

Similarly, comparison of community diversity and richness at the 3-week time point indicated differences only between the CON/VEH and DEF/VEH groups (Fig. 4f, phylogenetic diversity, *F*_2, 28_ = 7.893, *p* = 0.002; Chao1 richness, *F*_2, 28_ = 6.061, *p* = 0.007). Thus, these data indicate that social defeat-induced dysbiosis persisted for at least 3 weeks following stress exposure and the defeat-induced change in microbiome profile was not significantly altered by JB-1 treatment.

### The faecal metabolome is altered by exposure to chronic psychosocial stress and *L. rhamnosus* JB-1 treatment

Host and microbial metabolites play a contributory role in health and disease, including neural development and behaviour [16]. A total of 621 metabolites were detected in the faeces of mice; 70 were significantly altered by stress exposure (*q* < 0.1), many of which were associated with pathways previously predicted using *in silico* techniques: synthesis and metabolism of fatty acids, and tryptophan and tyrosine metabolism (Additional file 2: Table S3) [13]. Furthermore, 75 faecal metabolites were regulated by JB-1 treatment (Additional file 2: Table S4).

Previous work has demonstrated that JB-1 signals the brain via the vagus nerve [10, 22, 38]. To investigate signals that play a role in JB-1-driven vagal signalling, we explored for metabolites that were elevated exclusively in JB-1-treated stressed mice; however, no such metabolites were detected (*q* < 0.1).

**Fig. 5:**
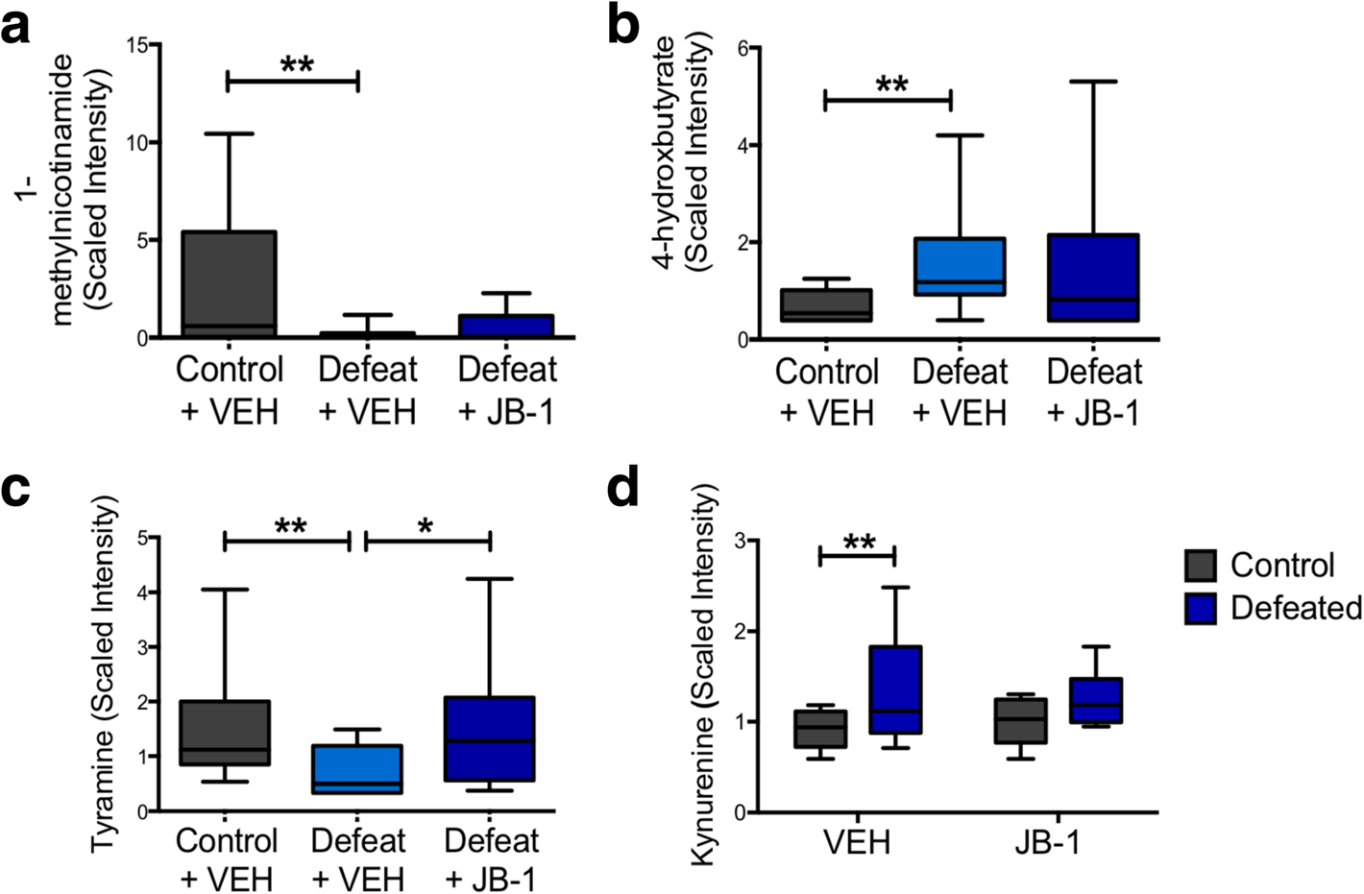
Effect of chronic stress and JB-1 treatment on the faecal metabolome. **a**–**d** Metabolites whose levels were altered by chronic stress but prevented in JB-1-treated mice (*n*: CON/VEH = 10, CON/JB-1 = 5, DEF/VEH = 10, DEF/JB-1 = 10). **a** 1-methylnicotinamide, **b** 4-hydroxybutryrate, **c** tyramine, **d** kynurenine. *p < 0.05, **p <0.01. Data are represented as median ± min/max

We investigated functional pathways that were altered by exposure to CSD, but not in JB-1-treated mice. This criterion (*q* < 0.1) yielded 15 metabolites (Fig. 5a, b; Additional file 2: Table S5), including 1-methylnicotinamide—a vitamin B3 derivative with anti-inflammatory effects [39]—and 4-hydroxybutryrate (GHB), a metabolite with neurotransmitter-like effects [40–42]. Other metabolites meeting this criterion include glutarate, *N*-acetylcitrulline, glycerate, lactobionate, 3-hydroxybutyrylcarnitine, 10-hydroxystearate, multiple metabolites derived from sphingolipid metabolism, alpha-muricholate, and lithocholate. However only for one metabolite that met this criteria, tyramine, a monoamine with sympathomimetic properties [43, 44], did the difference between DEF/VEH and DEF/JB-1 reach statistical significance (Fig. 5c).

Stress increased the levels of kynurenine in both groups of defeated mice (*F*_1, 31_ = 5.839, *p* = 0.022) (Fig. 5d). Planned post hoc analysis revealed significant differences between vehicle-treated defeated and control groups (*p* < 0.05) but none between JB-1-treated control and defeated mice (*p* > 0.05). Neither stress nor microbial treatment affected the kynurenine/tryptophan ratio—a sensitive estimate of cellular immunity [45, 46].

These data demonstrate that CSD alters the levels of various faecal metabolites, some of which possess immunomodulatory and neuroactive properties, and that JB-1 treatment may modulate some of these changes.

## Discussion

Using a validated model of chronic stress and depression [18, 19], we demonstrate, for the first time, the influence of a single orally administered bacteria strain, *Lactobacillus rhamnosus* JB-1, on behavioural deficits and systemic immune alterations caused by chronic exposure to a psychosocial stressor. While we observed no effects on baseline behaviour, JB-1 attenuated stress-induced behavioural deficits, including changes in sociability and anxiety-like behaviour, and prevented immunoregulatory alterations associated with the stress phenotype. Notably, the tempering of stress-induced changes occurred in the absence of any effects of treatment on stress-related disruptions in the microbiota, suggesting that JB-1 directly modulates gut-brain signalling pathways independently of the microbial community.

Following CSD, JB-1-treated stressed mice, as opposed to vehicle-treated, did not show avoidance of novel social stimuli, exhibited more frequent rearing behaviour in the OFT, and showed reduced aversion towards the light chamber (LDT). These data support the emerging literature suggesting that administration of specific bacterial strains decreases anxiety- and depressive-like behaviours [9, 10]. Indeed, we have previously demonstrated that chronic administration of JB-1 in Balb/c mice altered baseline levels of anxiety-like behaviour [10]. That the effects of JB-1 here were limited to deficits produced by chronic stress and not baseline behaviour (Fig. 2), may be indicative of intrinsic differences between Balb/c and C57BL/6 mice, the latter of which exhibit reduced apprehension, neophobia, and anxiety-like behaviour on baseline behavioural assays [47]. These mouse strain-specific effects may have implications for translational studies in humans, suggesting that, in keeping with a recent report [48], JB-1 would not be expected to have an anxiolytic effect in non-anxious individuals. Similarly, anti-depressants have very limited effects on healthy subjects [49].

In models of psychiatric conditions, repeated aggression and defeat lead to persistent conditioned submissive behaviour and aversion towards social stimuli [18, 50]. These behavioural manifestations bear similarity to symptoms of social withdrawal in depression and phobic avoidance of trauma-related stimuli in PTSD [51]. It is notable that the ameliorating effects of JB-1 on deficits in social behaviour were limited to interactions involving a non-threatening conspecific, while avoidance of the novel trauma-related stimulus was maintained. Previous research has suggested dissociation of social and non-social forms of anxiety-like behaviour [52]. For instance, treatment with a human commensal organism, *Bacteroides fragilis*, in a model of autism spectrum disorder attenuated deficits in anxiety-like behaviour, but did not affect sociability [31]. Our findings suggest that social anxiety may be further dissociated into discrete, differentially modulated behaviours expressed towards non-threatening versus threatening stimuli, the latter of which is experience-dependent [18, 19]. Thus, the disparate effects of JB-1 on behaviours expressed by defeated mice may be due to independent underlying neural circuitry. Such dissociable circuitry has been indicated by work on the stimulation of nucleus accumbens afferents, which alters behaviour towards a novel aggressor, but not anxiety-like behaviour [27]. This concept is further supported and emphasized in the current study given the recovery of anxiety-like behaviour but not of aggressor avoidance behaviour 3 weeks post-defeat (Fig. 2 h, i). In addressing neural mechanisms underlying the effect of microbial treatment on the expression of stress-related behaviours, we examined a limited number of genes related to the stress circuitry in the frontal cortex and hippocampus. While stress exposure reduced GABA receptor expression in the prefrontal cortex and glucocorticoid receptor expression in the hippocampus, there was no effect of microbe treatment on these measures. This contrasts the previously demonstrated effects of JB-1 administration on baseline expression of central GABA receptors in Balb/c mice [10]. While these results further emphasize the mouse strain-dependent effects of microbe exposure on gut-brain signalling, a more extensive assessment of additional neural pathways in multiple brain regions will be required to identify potential circuitry involved in JB-1-induced attenuation of stress-related behaviour.

Consistent with the immunomodulatory role of gut bacteria [15] and previous studies with JB-1 [24], microbial treatment influenced systemic changes in the CSD-induced immune phenotype. Social defeat increased the population of activated splenic DCs—a shift completely prevented by JB-1. Furthermore, treatment with the bacteria induced systemic expansion of Treg: a population that produces high levels of the anti-inflammatory cytokine, IL-10 [53]. Coordination between multiple host systems—and dysregulation thereof—likely contributes to the phenotypic changes in stress and related psychiatric conditions, during which systemic disruptions and allostatic load accumulate over extended periods of time. For instance, a pro-inflammatory milieu and a decrease in Tregs are commonly observed in severe stress and PTSD [17, 54] and form the central premise of the inflammation theory of depression [55]. Indeed, stress-induced trafficking of peripheral monocytes to the brain appears to play a crucial role in anxiety-like behaviour [56]. Disruption of the host-microbiota relationship during chronic stress may contribute to exaggerated inflammation and immune dysregulation and is associated with colitis and inflammatory bowel disease [11, 57]. The observed acute increase in the Treg population (Fig. 3a) [13] following stress may be a counteractive response to the pro-inflammatory shifts described in the literature upon stress induction [13, 56, 58]; such responses are a well-documented reaction to host inflammation in an attempt to restore homeostasis [59]. Although this natural allostatic mechanism does not prevent an inflammatory environment during maladaptive stress, JB-1-induced modulation of host-initiated immunoregulatory responses may be one mechanism contributing to the behavioural effects of the bacteria. Similar mechanisms were posited to explain the stress-mitigating effects of *Mycobacterium vaccae* immunization, which were demonstrated to depend on Tregs [11]. These data suggest that recruitment of immune pathways in bottom-up (gut-to-brain) signalling is important. The current study was limited to two immune cell lineages: dendritic cells and T cells. Clearly, additional immune cell types may make important contributions to gut-brain signalling. Future studies should include a broader assessment of the immune system and more detailed examination of microbiota-immune-neural coordination and dysregulation of these systems in stress.

It has been proposed widely that modifying the resident intestinal bacteria in disease can reverse microbial dysbiosis and restore homeostatic function [60, 61]. Such an approach is especially relevant given evidence of microbiota disruption in severe stress and psychiatric conditions and its association with adverse gastrointestinal outcomes [37]. Thus, we investigated whether the improved neurobehavioural phenotype due to microbial treatment was associated with alterations to the existing microbiota community. Prior to stress exposure, administration of JB-1 did not alter the profile of the microbiota—data that parallel observations in humans who were administered a different strain of *L. rhamnosus* [62]. Furthermore, in our study, microbial treatment did not prevent any of the shifts in the microbiota community due to stress exposure. JB-1 treatment also completely failed to restore the diversity and richness of the microbiota or correct the relative abundances of specific OTUs altered by stress. Thus, the neuroactive properties of the beneficial microbe may be mediated independently of restoring microbial community balance, and might be dependent on its functional activity and direct modulation of host signalling pathways.

Not unexpectedly, the stress-induced dybiosis was accompanied by a significant change in levels of various faecal metabolites, while, perhaps more surprisingly, JB-1 treatment alone significantly modulated the levels of 75 metabolites, many of which have immunomodulatory and neuroactive properties. While the source of these metabolites, host or microbe, cannot be identified, these observations suggest that JB-1 could alter the function of the existing gut microbiota without influencing composition. Most notably, the reduction in tyramine levels induced by CSD was the only metabolite change significantly inhibited by JB-1 treatment. Tyramine is a monoaminergic neuromodulator, acting as an agonist for trace amine-associated receptor 1 (TAAR1) [63]. Tyramine also causes the release of norepinephrine from sympathetic nerves, reversing re-uptake through the norepinephrine transporter and has been demonstrated to induce serotonin (5-HT) production by enterochromaffin cells [64]. Given that intestinal 5-HT [65] and catecholamines [66] have been proposed as mediators of microbe-gut-brain signalling via modulation of the enteric nervous system, the impact of luminal tyramine levels on the gut-brain axis may warrant further investigation. The current study focused on faecal metabolites, with the understanding that gut lumen metabolites acting at the level of the gut epithelium, enteroendocrine cells, and enteric nervous system may play a role in microbe-gut-brain signalling. However, future assessment of plasma metabolites may identify circulating factors, produced by gut microbes or induced in the host, which have more direct effects on the central nervous system.

One limitation of the current study is that we only assessed the faecal microbiota, and it is possible that JB-1 stabilized site-specific microbiota, for example, in the small intestine or specifically associated with the epithelium elsewhere, that are involved in gut-brain signalling. However, a direct action of JB-1 on gut-brain signalling is further supported by previous studies using in vivo and ex vivo models, demonstrating that it can directly or indirectly activate the vagus nerve and that an intact vagus is required to mediate the effects of this bacterium, at least on the baseline behaviour of Balb/c mice [22]. Collectively, these data suggest that JB-1, independently of changes in the microbiota, can recruit host signalling pathways, likely including vagal afferents that mediate the effects of the bacteria on severe CSD-induced neurobehavioural changes. Investigation of the role of the vagus in mediating microbe-induced modulation of behaviour in the CSD model is certainly warranted.

Although numerous studies have demonstrated the effect of environmental adversity on disruption of gut microbiota [11–13], there is very little evidence on the permanence of these changes in stress-related disorders or on whether microbial supplementation can facilitate the recovery of dysbiosis. A limited number of observations suggest a complex relationship between environmental factors and perturbations of the gut microbiota. Certain factors impart transient changes in the community, while others, for instance, antibiotic usage, leave behind a more persistent signature [67, 68]. Furthermore, factors such as birth delivery mode have marked effects on the microbiota community during early life that are no longer distinguishable in adulthood [69]. Our own observations suggest that stress-induced disruptions in the microbiota appear stable for a prolonged period following stress exposure. Examination of defeated mice 3 weeks following CSD revealed enduring structural changes in the faecal microbial community: defeated mice continued to show reduced diversity and richness in the variety of species represented while exhibiting broad-scale changes in overall composition and profile. The long-term stress-induced changes in the microbiome were not significantly altered with JB-1 treatment.

## Conclusions

There have been increasing efforts to understand how large-scale disruptions and dynamic shifts in gut microbiota can drive phenotypic changes and disease states [70]. This study represents a series of findings that further clarify the role of gut bacteria on neural function and behaviour. Although there continue to exist major gaps in our understanding of how disruptions in the microbiota contribute to neuropsychiatric conditions, the emerging theme underscores the intricate interactions between these systems in health and disease. While the current study did not delineate a mechanism of action of JB-1 in attenuating stress-induced behavioural changes, the results suggest that a more detailed investigation of immunomodulation and neural circuitry will likely provide valuable mechanistic insights. Furthermore, despite the complexity of the observed structural and functional changes in the microbial consortia, our data indicate that restoration of homeostasis can be facilitated using a single microbial strain. Given the diversity and inter-individual variability of the human gut microbiome [71–73], we propose that microbial-based interventions that bypass the microbiota to directly affect the host may possess greater therapeutic potential for the effective treatment of psychiatric conditions, or as an adjunct to current approaches.

## Additional files

Additional file 1: Supplementary information. (DOCX 30 kb)
Additional file 2: Supplementary Tables: Table S1. Statistical comparisons of Bray-Curtis distances between a priori groupings. Related to Figure 4. **Table S2**. discriminatory OTUs between undefeated vehicle- and JB-1-treated controls, and vehicle- and JB-1-treated defeated mice. Related to Figure 4. **Table S3**. fecal metabolites altered in response to stress. **Table S4**. Main effect of L. rhamnosus (JB-1) treatment on fecal metabolites. **Table S5**. fecal metabolites altered by stress (DEF/VEH vs CON/VEH) but unaffected in DEF/JB-1 vs CON/VEH. Related to Figure 5. **Table S6**. Primer sequences used for real time quantitative PCR. Related to Figure S2. (XLSX 83 kb)
Additional file 3: Figure S1. (A) Three-chamber sociability test paradigm. (B, C) Effect of chronic social defeat stress and JB-1 treatment on time spent in the center of the OFT and in the light chamber of the LDT. (D) Effect of JB-1 administration on detected levels of *Lactobacillus rhamnosus* cells in faecal samples. (E) Principle coordinates plots (*PCoA*) of Bray-Curtis distances from the average rarefied 16S rRNA data (*n* = 999 rarefactions, 52,182 reads/sample) indicate no effect of JB-1 treatment after 18 days, prior to initiation of chronic social defeat stress. (F) Related to Fig. 4e: principle coordinates plots (PCoA) of Bray-Curtis distances from the average rarefied 16S rRNA data (*n* = 999 rarefactions, 44,648 reads/sample) 3 weeks after stressor and treatment cessation indicate a persistent significant effect of social defeat on group clustering (*p* = 0.022), but no difference between the control and DEF/JB-1 groups. (PDF 206 kb)
Additional file 4: Figure S2. (A–J). Effect of chronic social defeat stress and JB-1 treatment on gene expression levels in the frontal cortex (*n* = 5–13/group) and the hippocampus (*n* = 4–8/group). (PDF 258 kb)
