# Supplementary material for: Oral treatment with Lactobacillus rhamnosus attenuates behavioural deficits and immune changes in chronic social stress
Source: BMC Med. 2017 Jan 11;15:7. doi: 10.1186/s12916-016-0771-7 (PMC5225647; doi:10.1186/s12916-016-0771-7)
Supplement: Additional file 1: — Supplementary information. (DOCX 30 kb) [file 12916_2016_771_MOESM1_ESM.docx]

Additional file 1

***Supplemental Information***

**Preparation and treatment with *Lactobacillus rhamnosus* (JB-1).** *Lactobacillus rhamnosus* (JB-1) ^TM^ bacteria were prepared as described previously (1). Briefly, bacteria from stock were suspended in tubes filled with Man-Rogosa-Sharpe (MRS) medium for 48-h under anaerobic conditions. Following this, bacteria were harvested, washed with sterile phosphate buffered saline (PBS) to the desired concentration, re-suspended in MRS broth, and stored at -80°C in 1ml aliquots at 10^10^ colony-forming units (CFUs)/ml.

**Social Defeat.** During the final 10 days of oral treatment with *L. rhamnosus* (JB-1) or vehicle (Fig. 1)*,* chronic social defeat (CSD) procedures were initiated daily over 10 consecutive days, as previously described (2). During each defeat session, intruder C57BL/6 mice were allowed to interact for 5 minutes with a novel resident CD-1 mouse. Defeated mice were observed to ensure the expression of subordinate posturing. For 24 hours after each defeat, mice were house in the same cage as the aggressor across a perforated Plexiglas divider. Paired control mice were housed on either side of a Plexiglas divider while preventing any physical contact. Upon completion of the final CSD session, the animal underwent behavioural testing as described previously (Fig. 1) (3).

***Open Field Test (OFT).*** Testing was carried out under dim-light conditions in the dark phase of the LD cycle, one day after exposure to the final defeat session (Fig. 1). After one-hour habituation to the testing room, mice were singly placed in an 18 x 38 cm Plexiglas enclosure for 30 minutes. Distance traveled, rearing count, and time spent in the center of the field were recorded via photo beam sensors outfitted around the arena (Motor Monitor; Kinder Scientific). Equipment was cleaned between each test.

***Light-Dark box (LD) Test.***  On day 2 following the final defeat session, testing was carried out in the dark phase of the LD cycle under dim-light conditions. After one-hour habituation to the testing room, mice were singly placed for a period of 10 minutes into an 18 x 38 cm clear Plexiglas enclosure containing a black insert at one end. Kinderscientific Motor Monitor software was used to record time in the light zone and entries into the light zone. The equipment was cleaned between each test.

***Three-Chamber Sociability Test.*** Tests were conducted 3 days after the final defeat session, during the light phase of the LD cycle, following a 30-minute habituation period in the testing room. The testing apparatus was a Plexiglas box with three chambers (Fig. S1A), each measuring 24.5 cm L x 44 cm W x 30 cm H. The dividing chamber walls possessed openings to allow mice access to each chamber. During the habituation phase, the test mouse was placed in the center chamber—with access to side chambers obstructed—and allowed to freely explore for 5 minutes. Following this, an unfamiliar sex- and strain-matched conspecific (stranger) was placed within a round, wire cup in one of the side chambers (social chamber). An identical inverted wire cup containing a novel object was placed in the other side chamber (non-social chamber). During the sociability phase, the test mouse was placed in the center chamber and allowed to freely explore all three chambers for 10 minutes. Distanced moved and time spent in each chamber was recorded by a video camera (EthoVision XT; Noldus). The equipment was cleaned between test mice.

***Aggressor Approach-Avoidance Test.*** To assess social behaviour towards a novel social target resembling the social defeat aggressors (2), mice were placed in a 24.5 cm x 44 cm arena for two sessions of 150s each on day 3 post-stressor (immediately following the three-chamber sociability test). EthoVision XT (Noldus) was used to divide the virtual map of the arena into two equal halves—interaction zone and non-interaction zone. During the first session (aggressor absent), an empty wire mesh cage was placed at one end of the arena, in the interaction zone (Fig. 2D). During the second session (aggressor present), a novel CD1 aggressor was placed under the wired cage. Between the two sessions, the experimental mouse was placed in its home cage for one minute during setup of the ‘aggressor present’ session. Time spent in the interaction zone during the aggressor absent and aggressor present sessions was calculated for each mouse. Only susceptible mice (social interaction ratio <1, time spent in interaction zone during aggressor presence/time spent in interaction zone during aggressor absence) were specifically selected from the cohort of defeated mice for all experiments.

**16s rRNA Analysis of Bacterial Composition.** One day before the first defeat session (i.e. following the 18^th^ day of treatment with *L. rhamnosus JB-1*), one day following the final defeat session (i.e. following the final day of JB-1 treatment), and 3 weeks after the final defeat session, fecal pellets were collected and stored at -80^o^C for molecular analysis of microbiota**.** DNA extraction was carried out as previously described (3). Bacterial community profiling of 16S rRNA gene was carried out using a modified bar coded Illumina sequencing method (4). Paired end reads of the V3 region were performed using the 341F and 518R primers (5). 250 nt paired-end sequencing was carried out on a MiSeq Illumina sequencer as per manufacturer’s instructions. Sequencing was carried out on a MiSeq Illumina sequencer in the McMaster Genome Center (McMaster University).

The MiSeq data was processed by an in-house bioinformatics pipeline (6) that incorporates quality filtering. Sequencing results produced 7406 operational taxonomic units (OTUs), and a minimum, maximum, and median of 7923, 194481, and 118315 reads/sample respectively. Using QIIME (7), singletons were excluded and OTU tables underwent ten repeated rarefactions at multiple sequencing depths to enable equal reads across samples. For alpha diversity analysis, Chao1 and Phylogenetic Diversity metrics were recruited using the alpha diversity workflow script. Each metric was implemented using the same number of sequences as the most indigent sample. Mann Whitney *U* or two-way ANOVA tests were used to assess statistical significance of measures derived from alpha diversity metrics. For beta-diversity analyses, Jackknife resampling at a sequencing depth equal to 80% of the most indigent sample was used to generate Bray-Curtis distance matrices. (Dis)similarity between the group microbiota profiles was assessed through multiple *a priori* comparisons, using the Monte Carlo Permutation Procedure (MCPP) (999 permutations) and Bonferroni-corrected non-parametric t-tests. The Kruskal-Wallis one-way ANOVA or the Mann Whitney U test, followed by the Benjamini-Hochberg correction for multiple comparisons (False Discovery Rate < 0.05) was used to analyze differential abundance of OTUs in groups using data first rarefied to even sequencing depth and then filtered to eliminate OTUs observed in fewer than 25% of the samples.

**Detection of faecal *L. rhamnosus* levels.** Fecal content in JB1 was estimated using qPCR of the 16S rRNA gene for *L. rhamnosus*. DNA was extracted from feces using a QIAamp DNA Stool Mini Kit (Qiagen, Hilden, Germany) according to the manufacturer’s instructions, and including a bead-beating step. Real-time polymerase chain reaction (PCR) was performed with a StepOnePlus Real-Time PCR System and software (Applied Biosystems, Den Ijssel, The Netherlands) using SYBR Green (Applied Biosystems) for detection. Primers sequences were as followed: Lr1 GTGCTTGCATCTTGATTTAATTTT and Lr2 TGCGGTTCTTGGATCTATGCG as reported by Furet *et al*. (8) (annealing temperature of 50°C). All samples were run in duplicate in a single 96-well reaction plate. The purity of the amplified product was verified by analyzing the melting curve performed at the end of amplification. Quantification was achieved through a standard curve generated using DNA isolated from a culture-quantified aliquot of JB1 following the same extraction procedure.

**Metabolomics.** One day following the final defeat session (i.e. following the final day of JB-1 treatment), fecal pellets were collected and stored at -80^o^C**.** Samples were prepared using the automated MicroLab STAR® system from Hamilton Company and analyzed by Metabolon, Inc. using Ultrahigh Performance Liquid Chromatography-Tandem Mass Spectroscopy (UPLC-MS/MS) platforms. Samples were lyophilized and an identical mass equivalent was extracted and processed for the platform*.* Proteins were precipitated with methanol under vigorous shaking for 2 min (Glen Mills GenoGrinder 2000) followed by centrifugation. Samples were placed briefly on a TurboVap® (Zymark) to remove the organic solvent. The sample extracts were stored overnight under nitrogen before preparation for analysis, when the extract was dried then reconstituted in compatible acidic and basic solvents. Each reconstitution solvent contained a series of standards at fixed concentrations to ensure injection and chromatographic consistency. All methods utilized a Waters ACQUITY ultra-performance liquid chromatography (UPLC) and a Thermo Scientific Q-Exactive high resolution/accurate mass spectrometer interfaced with a heated electrospray ionization (HESI-II) source and Orbitrap mass analyzer operated at 35,000 mass resolution.

**References**

1. Bravo J a., Forsythe P, Chew M V., Escaravage E, Savignac HM, Dinan TG, *et al.* (2011): Ingestion of Lactobacillus strain regulates emotional behavior and central GABA receptor expression in a mouse via the vagus nerve. *Proc Natl Acad Sci*. 108: 16050–16055.

2. Berton O, McClung C a, Dileone RJ, Krishnan V, Renthal W, Russo SJ, *et al.* (2006): Essential role of BDNF in the mesolimbic dopamine pathway in social defeat stress. *Science*. 311: 864–868.

3. Bharwani A, Mian MF, Foster JA, Surette MG, Bienenstock J, Forsythe P (2016): Structural & functional consequences of chronic psychosocial stress on the microbiome & host. *Psychoneuroendocrinology*. 63: 217–227.

4. Bartram AK, Lynch MDJ, Stearns JC, Moreno-Hagelsieb G, Neufeld JD (2011): Generation of multimillion-sequence 16S rRNA gene libraries from complex microbial communities by assembling paired-end Illumina reads. *Appl Environ Microbiol*. 77: 3846–3852.

5. Muyzer G, De Waal EC, Uitterlinden AG (1993): Profiling of complex microbial populations by denaturing gradient gel electrophoresis analysis of polymerase chain reaction-amplified genes coding for 16S rRNA. *Appl Environ Microbiol*. 59: 695–700.

6. Whelan FJ, Verschoor CP, Stearns JC, Rossi L, Luinstra K, Loeb M, *et al.* (2014): The loss of topography in the microbial communities of the upper respiratory tract in the elderly. *Ann Am Thorac Soc*. 11: 513–521.

7. Caporaso JG, Kuczynski J, Stombaugh J, Bittinger K, Bushman FD, Costello EK, *et al.* (2010): QIIME allows analysis of high- throughput community sequencing data. *Nat Methods*. 7: 335–336.

8. Furet J-P, Quénée P, Tailliez P (2004): Molecular quantification of lactic acid bacteria in fermented milk products using real-time quantitative PCR. *Int J Food Microbiol*. 97: 197–207.
