# Supplementary figures and images for: Oral treatment with Lactobacillus rhamnosus attenuates behavioural deficits and immune changes in chronic social stress
Source: BMC Med. 2017 Jan 11;15:7. doi: 10.1186/s12916-016-0771-7 (PMC5225647; doi:10.1186/s12916-016-0771-7)

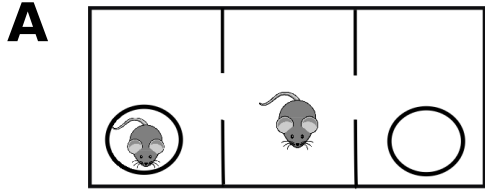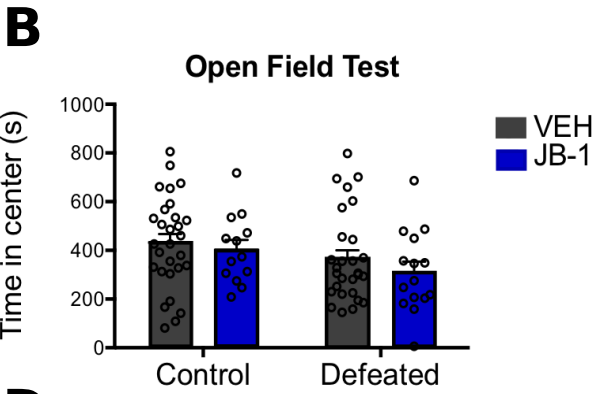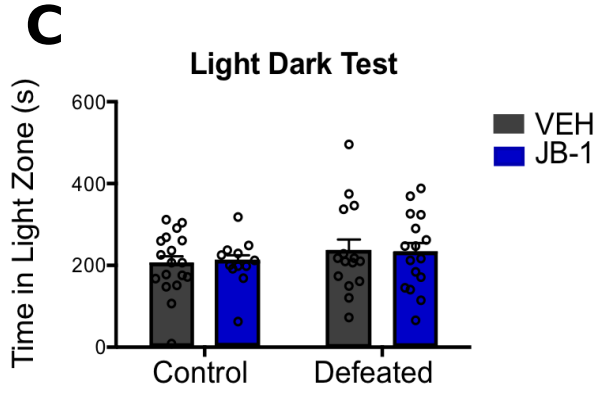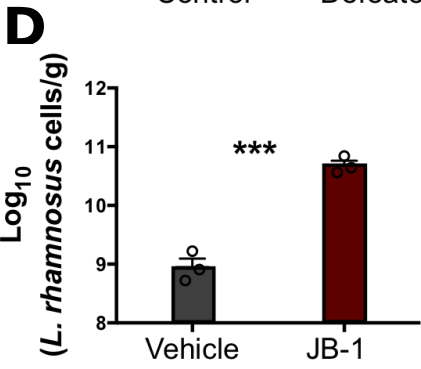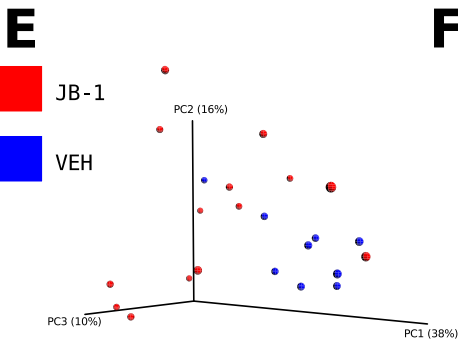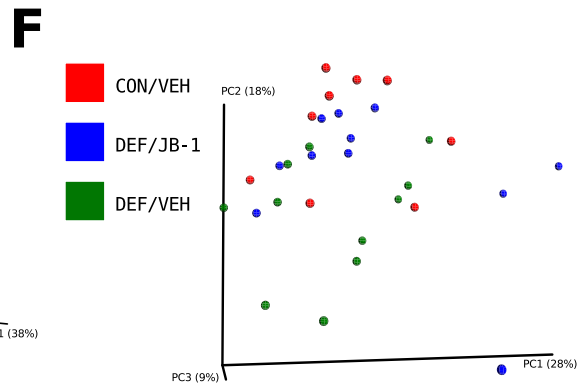

Supplement: Additional file 3: Figure S1. — (A) Three-chamber sociability test paradigm. (B, C) Effect of chronic social defeat stress and JB-1 treatment on time spent in the center of the OFT and in the light chamber of the LDT. (D) Effect of JB-1 administration on detected levels of Lactobacillus rhamnosus cells in faecal samples. (E) Principle coordinates plots (PCoA) of Bray-Curtis distances from the average rarefied 16S rRNA data (n = 999 rarefactions, 52,182 reads/sample) indicate no effect of JB-1 treatment after 18 days, prior to initiation of chronic social defeat stress. (F) Related to Fig. 4e: principle coordinates plots (PCoA) of Bray-Curtis distances from the average rarefied 16S rRNA data (n = 999 rarefactions, 44,648 reads/sample) 3 weeks after stressor and treatment cessation indicate a persistent significant effect of social defeat on group clustering (p = 0.022), but no difference between the control and DEF/JB-1 groups. (PDF 206 kb) [file 12916_2016_771_MOESM3_ESM.pdf]

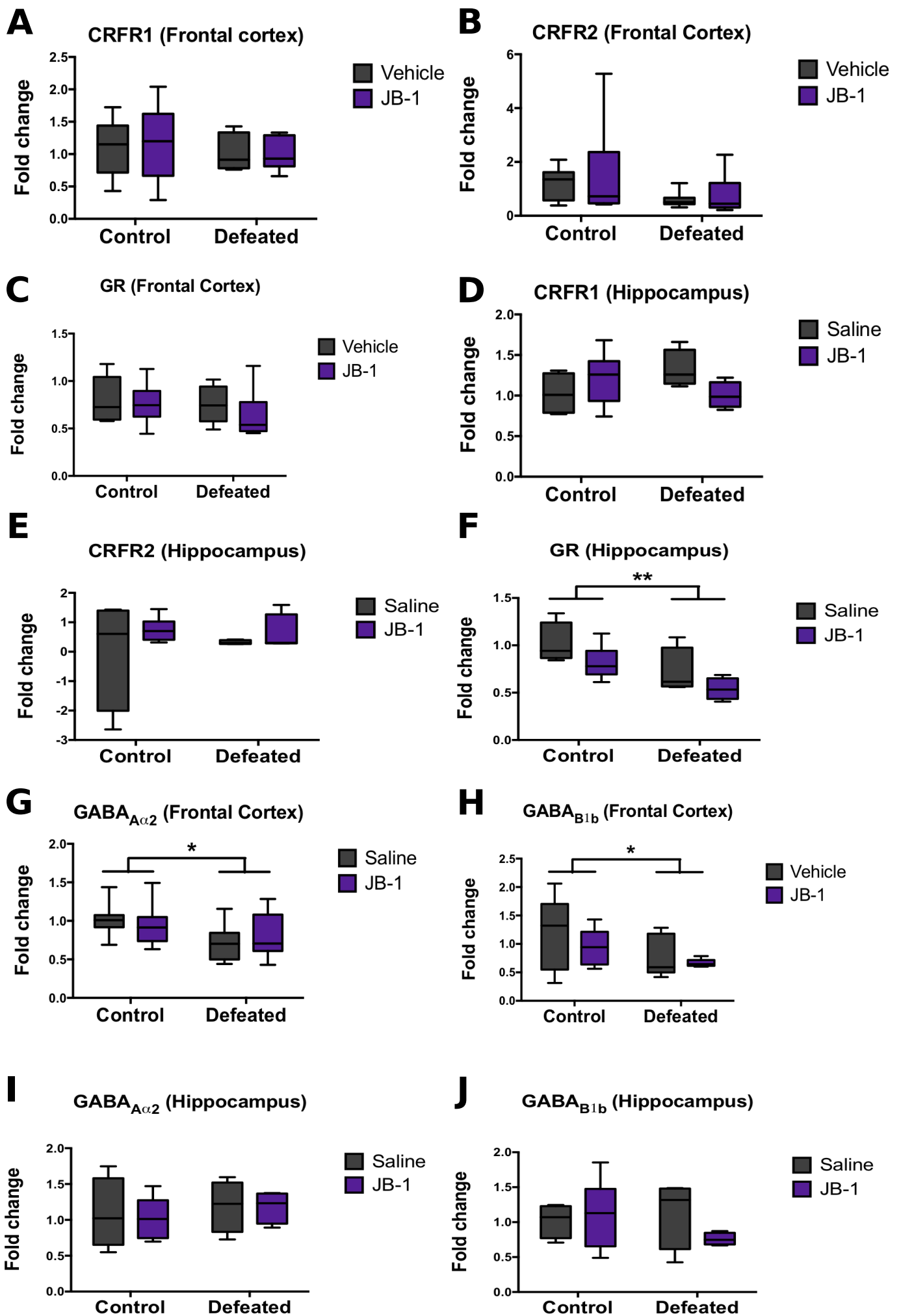

Supplement: Additional file 4: Figure S2. — (A–J). Effect of chronic social defeat stress and JB-1 treatment on gene expression levels in the frontal cortex (n = 5–13/group) and the hippocampus (n = 4–8/group). (PDF 258 kb) [file 12916_2016_771_MOESM4_ESM.pdf]
